# Adaptive Anchoring Model: How Static and Dynamic Presentations of Time Series Influence Judgments and Predictions

**DOI:** 10.1111/cogs.12476

**Published:** 2017-04-06

**Authors:** Petko Kusev, Paul van Schaik, Krasimira Tsaneva‐Atanasova, Asgeir Juliusson, Nick Chater

**Affiliations:** ^1^ Department of Psychology Kingston University London; ^2^ School of Social Sciences, Business & Law Teesside University; ^3^ Department of Mathematics University of Exeter; ^4^ Department of Psychology City University London; ^5^ Behavioural Science Group Warwick Business School

**Keywords:** Judgment, Prediction, Experience, Description, Trend, Behavioral forecasting

## Abstract

When attempting to predict future events, people commonly rely on historical data. One psychological characteristic of judgmental forecasting of time series, established by research, is that when people make forecasts from series, they tend to underestimate future values for upward trends and overestimate them for downward ones, so‐called trend‐damping (modeled by anchoring on, and insufficient adjustment from, the average of recent time series values). Events in a time series can be experienced sequentially (dynamic mode), or they can also be retrospectively viewed simultaneously (static mode), not experienced individually in real time. In one experiment, we studied the influence of presentation mode (dynamic and static) on two sorts of judgment: (a) predictions of the next event (forecast) and (b) estimation of the average value of all the events in the presented series (average estimation). Participants' responses in dynamic mode were anchored on more recent events than in static mode for all types of judgment but with different consequences; hence, dynamic presentation improved prediction accuracy, but not estimation. These results are not anticipated by existing theoretical accounts; we develop and present an agent‐based model—the adaptive anchoring model (ADAM)—to account for the difference between processing sequences of dynamically and statically presented stimuli (visually presented data). ADAM captures how variation in presentation mode produces variation in responses (and the accuracy of these responses) in both forecasting and judgment tasks. ADAM's model predictions for the forecasting and judgment tasks fit better with the response data than a linear‐regression time series model. Moreover, ADAM outperformed autoregressive‐integrated‐moving‐average (ARIMA) and exponential‐smoothing models, while neither of these models accounts for people's responses on the average estimation task.

## 1 Introduction

Recent research on decision making from experience suggests that the manner in which people acquire information affects how they respond to it. Theorists claimed that how people make risky decisions depends on whether they learned about the decision outcome values and their likelihoods either by repeatedly sampling the options and experiencing their outcomes, or via summary descriptions of the outcome values and likelihoods (Hertwig, Barron, Weber, & Erev, 2004; Hertwig & Erev, 2009; Hertwig & Pleskac, 2010; Kusev, van Schaik, Alzahrani, Lonigro, & Purser, 2016; Kusev, van Schaik, Ayton, Dent, & Chater, 2009). Their findings in support of this claim are significant because, although in life many choices are made without the benefit of descriptions of the likelihoods of the possible outcomes, nearly all the experimental research studying risky decision making has evaluated how people respond to described decisions^1^ (De Martino, Kumaran, Seymour, & Dolan, 2006; Tom, Fox, Trepel, & Poldrack, 2007).

Events in a time series can be experienced sequentially (dynamic mode), or they can also be viewed simultaneously (static mode), not experienced individually in real time. Outside the laboratory, decisions are usually based on experience; often people learn about the likelihoods of decision outcomes through repeatedly making choices and experiencing the outcomes. Some of the differences between experience‐based and description‐based decision making also apply to some forms of judgment. For example, sometimes people attempting to forecast from a time series of events will examine a complete series of historical data (e.g., recent sales performance) and attempt to predict the next event. Commonly, however, forecasters live through the sequence of events and, rather than inspecting a complete and static sequence, will experience a time series of events dynamically as they occur over time. In the latter case, as in learning about decisions from experience, the events in the time series are experienced sequentially in the context provided by subsequent events. In the former case, the events in the time series are not experienced individually over time, but they are reviewed retrospectively and can immediately be viewed holistically such that any overall pattern will be immediately apparent, as with learning about decisions from descriptions. Nevertheless, in both situations forecasters refer to exactly the same data points to predict the next event.

As decisions from experience differ from decisions from description, this plainly raises the issue as to how judgment from experience might differ from judgment from description; specifically, does the mode of encountering time series influence judgment as it does decision making? One psychological characteristic of judgmental forecasting of time series established by research is that when people make forecasts from series, they tend to underestimate future values for upward trends and overestimate them for downward ones, so‐called *trend‐damping* (Andreassen & Kraus, 1990; Bolger & Harvey, 1993; Eggleton, 1982; Keren, 1983; Lawrence & Makridakis, 1989; Reimers & Harvey, 2011; Sanders, 1992; Wagenaar & Sagaria, 1975; Wagenaar & Timmers, 1978). Research has also offered a number of theoretical explanations as to why trend‐damping may occur (Lawrence, Goodwin, O'Connor, & Önkal, 2006; Lawrence & O'Connor, 1992) and demonstrated that trend‐damping can be modeled (a) by anchoring on, and (insufficient) adjustment from, the average of recent time series values (Tversky & Kahneman, 1974) or (b) by contextual adaptation to features of the environment—with steeper trends causing trend‐damping and shallower trends leading to anti‐damping in behavioral forecasts (Harvey & Reimers, 2013). Here, we investigate whether both judgments of the average and judgmental forecasting of time series are affected by variation in the way in which people experience information, when either retrospectively reviewing a time series or experiencing it one event at a time. Given that trend‐damping reflects some psychological strategy, we aimed to determine if and how it varies across different ways of encountering a time series. Previous research has demonstrated that judgments are sensitive to contextual properties of experienced events (Kusev, Tsaneva‐Atanasova, van Schaik, & Chater, 2012; Kusev et al., 2011); experiencing a sequence of events serially (dynamically) one at a time necessarily draws attention to each individual event. Consequently, this may increase the tendency to anchor on the most salient events for experienced sequences more than for described sequences.

## 2 Predictions and model development

A commonly expressed view in memory and cognition research is that the representation of events stored in memory is highly dependent on the mode of psychological processing used in encoding the events (Bogen & Gazzaniga, 1965; Dunn, 1985; Dunn & Reddix, 1991; Levy, Trevarthen, & Sperry, 1972; Levy‐Agresti & Sperry, 1968; Tucker, 1981). One mode is traditionally considered to be analytic and logical in its processing (e.g., processing individual events, dynamically, one at a time), while the other mode processes information in a more holistic or Gestalt manner (e.g., processing the events, statically, at once) (Dunn, 1985; Dunn & Reddix, 1991; Levy et al., 1972; Levy‐Agresti & Sperry, 1968; Masuda & Nisbett, 2001; Nisbett & Miyamoto, 2006; Nisbett, Peng, Choi, & Norenzayan, 2001; Tucker, 1981; Van Belle, de Graef, Verfaillie, Busigny, & Rossion, 2010). Accordingly, we propose that forecasters, in their predictions, rely on a small sample of recent events (e.g., the most recent event from dynamically experienced sequences) or “average” representations of the whole time series from statically experienced sequences (e.g., Deese & Kaufman, 1957; Harvey & Reimers, 2013; Murdock, 1962; Postman & Phillips, 1965). However, neither empirical nor theoretical research provides evidence as to whether dynamically experienced sequences facilitate a general cognitive and behavioral advantage.

It is plausible that forecasting accuracy is enhanced by dynamically experienced sequences of events (where success accuracy is informed by the most recent event). Specifically, where events are presented with dynamic sequences (e.g., self‐paced value‐by‐value presentation of a series with all previous values visible at all times or self‐paced value‐by‐value presentation of a series with the last value only visible at any one time), forecasting accuracy may be induced, drawing the respondents' attention to each individual event; hence, anchoring on the most recent/salient event occurs. In contrast, where events are presented with static sequences (one simultaneous presentation of all values of a series), forecasting errors may be induced (drawing the respondents' attention on the “average” event with insufficient adjustment to the most recent event). This assumption is novel, intriguing, intuitive, and worth exploring.

However, presentation by way of either dynamic or static trends (time series) does not only influence forecasting. Accordingly, in this article we further explored the influence of two sorts of judgment: (a) predictions of the next event (forecast—where the focus is on the next event in time series), and (b) estimation of the average value of all the events in the presented series (average estimation—where the focus is on the “average” event in time series). It is likely that participants' responses in dynamic mode are anchored on more recent events than in static mode for all types of judgment (forecasting and average estimations), but with different consequences on judgment accuracy: dynamic presentation would improve prediction accuracy, but not estimation.

Accordingly, we assume that behavioral differences in the judgment of information may emerge as a result of the presentation mode of experienced (dynamic or static) sequences of perceptual information in the task and type of judgment (forecasting or estimation). In the present article, for the purpose of developing a unified model to account for the different modes of presentation, the terms *dynamic* and *static* will be used operationally to describe different presentations modes that are experienced, based on statically presented (described) and dynamically presented (experienced) sequences.

To comprehensively account for the effect of presentation mode for two types of judgment, we developed an agent‐based model—the adaptive anchoring model (ADAM), inspired by the memory‐based scaling model (Petrov & Anderson, 2005). Anchoring in the model is scaled via a stimulus parameter representing the type of experience and judgment task. We note that this is not a free parameter but attains a rather well‐defined value that accounts for the context of information presentation. The model is adaptive in the sense that it takes into account the extent of match between judgment task and experience type. In particular, in constructing the model we aim to account for people's responses in forecasting and judgment tasks across combinations of different presentation modes, trend directions, and trend consistency. First, presentation mode (described and experienced) will have an effect on participants' response: With static presentation the series average will be strongly weighted in the response (“average” event), but with dynamic presentation the last trend value will be strongly weighted in the response. Second, the judgment task would have an effect: In the forecasting task the last trend value will be strongly weighted in the response, but in the judgment task the series average will be strongly weighted in the response. Hence, we include in the model a predictor (parameter) that accounts for the distribution of weights, depending on the context of the data presentation and judgment task.

In our model, we use a single idealized item stimulus *S*_*i*_, which depends on the type of the experience: *i *= *s* (static), *i *= *dt* (dynamic with trend), or *i *= *dc* (dynamic with only the current value presented). In the case of static experience, *S*_*s*_ = 0.35 (the normalized mean of the experimental sequence), whereas in the case of dynamic experience *S*_*dt*_ = *S*_*dc*_ = normalized last element of the experimental sequence with range [0; 1]. The task effect in the model is represented by an idealized task item (*T*_*k*_), which depends on the experimental task, *k *= *j* (judging average) or *f* (forecasting). In particular, in the case of judgment *T*_*j*_ = 0.35 (the normalized mean of the experimental sequence), and in the case of dynamic experimental setting *T*_*f*_ = normalized last element of the experimental sequence.

Next, the similarity between the stimulus and the task is assumed to depend exponentially on the distance between them (Myung, Pitt, & Navarro, 2007). To take account of the trend in the model, we do not use the absolute value of the distance between stimulus and task item; we also normalize the values of the stimulus and task items such that *S*_*i*_ and *T*_*k*_ ∈ (0, 1). Hence, the similarity weight (*SW*_*ik*_) depends on the distance between an idealized item stimulus *S*_*i*_ and an idealized task item (*T*_*k*_). Specifically, we calculate the similarity weight using the following exponential representation:

(1) $$SW_{ik}=\exp\left(-\left(S_{i}-T_{k}\right)\right)$$

In addition, we take into account an experience‐dependent anchor, *EA*_*i*_, *i *= *s* (static) *i *= *dt* (historic‐dynamic, i.e., dynamic with trend), or *i *= *dc* (momentary‐dynamic, i.e., dynamic with current value only). In the case of static experience *EA*_*s*_ = 3,500 (the mean of the experimental sequence), whereas in the case of dynamic experience *EA*_*dt*_ = *EA*_*dc*_ = last element of the experimental sequence.

Furthermore, the similarity weight scales the magnitude of the behavioral response depending on the distance between the stimulus and the task target; in other words, it represents critical factors that may act in relation to the position of the stimulus. The stimuli in our experiments are time series bars with heights (representing quantities) and the task of the observers is to judge the average and forecast the next trend value (both tasks using response bars with heights). This is a similar version to some of the abstractions implicit in multidimensional scaling (Schiffman, Reynolds, & Young, 1981) and distance‐based similarity metrics (Nosofsky, 1992).

Finally, as suggested by the memory‐based scaling model (Petrov & Anderson, 2005), we take into account perceptual noise (to account for individual perceptual variability among participants). Hence, the behavioral response (*BR*_*ik*_) in the model is a Gaussian random variable whose mean and variance depend on the stimulus‐task similarity weight, *SW*_*ik*_, as well as the experience‐dependent anchor, *EA*_*i*_, and is given by the following product:

(2) $$BR_{ik}=SW_{ik}\times EA_{i}\times \left(1+k_{p}x_{p}\right),$$

where *k*_*p*_ is a dimensionless coefficient of proportionality and *x*_*p*_ is a random variable with zero mean and unit variance (Petrov & Anderson, 2005). We note that Eq. (2) can be considered a stochastic version of the linear regression equations used in previous research on forecasting and judgment where the anchor point depends on specific properties of the time series used in the experiments (Lawrence & O'Connor, 1992, 1995). However, our model takes into account the similarity between task and type of experience.

## 3 Experiment

We expect that human forecasting and average estimation are informed by the most recent event from dynamically experienced sequences (dynamic mode) or “average” representations of the whole time series from statically experienced sequences (static mode), with different consequences on judgment accuracy. Specifically, we predict a fourfold pattern of judgment accuracy: (a) enhanced forecasting accuracy with dynamic mode (where success accuracy will be informed by the most recent event), (b) induced average estimation errors with dynamic mode (recency), (c) induced forecasting errors with static mode (drawing the respondents' attention on the “average” event with insufficient adjustment to the most recent event), and (d) enhanced average estimation accuracy with static mode (one simultaneous presentation of all values of a series).

Accordingly, an experiment was designed to establish the effects of presentation mode of experiencing a time series and judgment tasks on behavioral response, and to evaluate the difference between the behavioral response data and model predictions from ADAM, linear regression, autoregressive‐integrated‐moving‐average (ARIMA), and exponential smoothing models. Two sorts of judgment were studied: (a) predictions of the next event (*forecasting*), and (b) estimation of the average value of all the events in the presented series (*average estimation*). We chose these two judgment tasks as they are somewhat disparate; plausibly, they will invoke diverse processing strategies requiring differential attention to features of the time series (trends, distribution, volatility) that may be differentially influenced by the mode of experiencing time series.

### 3.1 Method

#### 3.1.1 Participants

One thousand six hundred and twenty participants (876 female; *M*_age_ = 42, *SD* = 13) were recruited through a recruitment service of online survey panels. They took part individually and received a payment of £1. All participants were treated in accordance with the British Psychological Society's code of human research ethics.

#### 3.1.2 Stimuli and equipment

**Figure 1:**
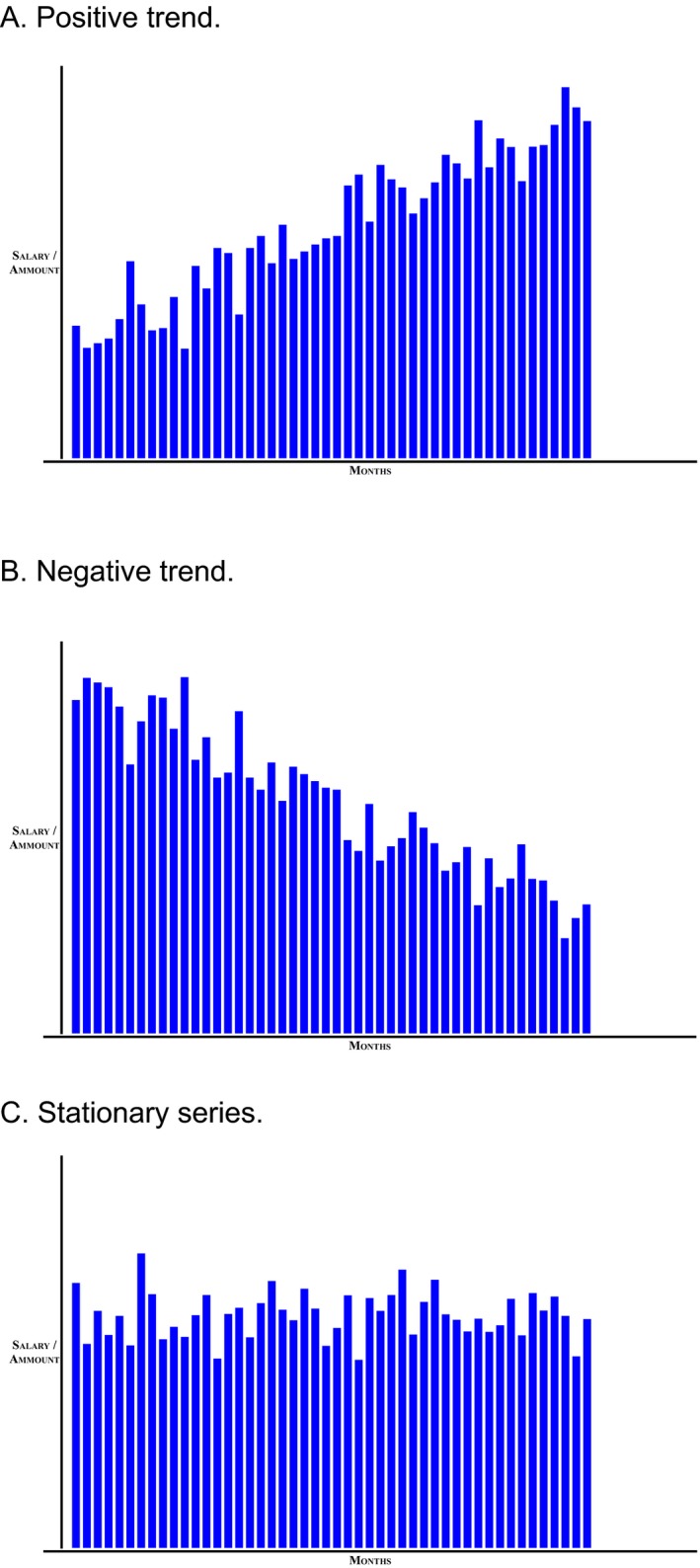
Time series used in the experiment.

An interactive computer program for judgments and forecasting was developed and used. Three time series (linear positive trend with superimposed noise, linear negative trend with superimposed noise and stationary noise only; see Fig. 1A–C) of monetary values were generated. The three time series can be described with Eqs. (3), (4), (5), respectively:

(3) Y(t)=3,000+21.28×t+n.

(4) Y(t)=4,000−21.28×t+n.

(5) Y(t)=3,500+n,

where *t* = 1, …, 48, and *n* followed a normal distribution with mean = 0 and variance = 10.

#### 3.1.3 Design and procedure

A 3 × 3 × 2 independent‐measures experimental design was used. The first independent variable was *trend direction*; this was positive linear trend with superimposed Gaussian noise, negative trend with superimposed Gaussian noise, or stationary series (Gaussian noise only). The second independent variable was *presentation mode*; this was historic‐dynamic (self‐paced value‐by‐value presentation of a series with all previous values visible at all times), momentary‐dynamic (self‐paced value‐by‐value presentation of a series with only the last value visible at any one time), or static (one simultaneous presentation of all values of a series). The third independent variable was *trend consistency*; this was consistent or inconsistent. In consistent trends, 48 data points were presented, according to Eqs. (3), (4), (5). In inconsistent trends, 49 data points were presented; after 48 points according to Eqs. (3), (4), (5), the mean value of 3,500 followed, producing an inconsistent continuation of positive and negative trends. Participants were asked to make only two judgments (no multiple forecasts and estimations) from the time series: a prediction of the next event, and a judgment of the average value in the series after being presented with the entire 48 or 49 data points.

Accordingly, the first dependent variable was forecasting error. This was relative to the next predicted value from the appropriate regression model—(3), (4), or (5) for consistent trends and with adjusted model parameter values to account for inconsistent trends with an additional 49th data point. The second dependent variable was error in the judged average value (relative to the true mean, which was the same for all series).

Participants were told that the time series represented the average monthly earnings (in pounds sterling) of employees in one company over 48 or 49 months. In the static presentation the whole series was displayed at once (with no behavioral dynamic task engagement). In contrast, tasks with dynamic presentation required participants to click a button (labeled “*Next Month*”) to observe each month's salaries, with previous values remaining on the screen (in historic‐dynamic presentation) or not (in momentary‐dynamic presentation). Therefore, all values of the series were presented in both the static and dynamic conditions before any type of judgment was required to be made by the participants. In each presentation condition, while the whole series (for static and historic‐dynamic presentation) or the final value (for momentary‐dynamic presentation) was displayed, participants had to (a) predict the next value in the series (by clicking at their chosen position on a vertical line), and (b) estimate the average salary over the presented 48 or 49 months (again, by clicking). These two tasks (performed only once) were presented in random order for each participant and without training.

### 3.2 Results and discussion

We first present an exploratory analysis of the first two dependent variables, before analyzing each of the two dependent variables in detail. We found that the mode of presentation (historic‐dynamic, momentary‐dynamic, or static) affected judgment of both dependent variables (error of behavioral forecasting and average estimation), but the effect was moderated by trend direction and trend consistency.

#### 3.2.1 Exploratory analysis

**Figure 2:**
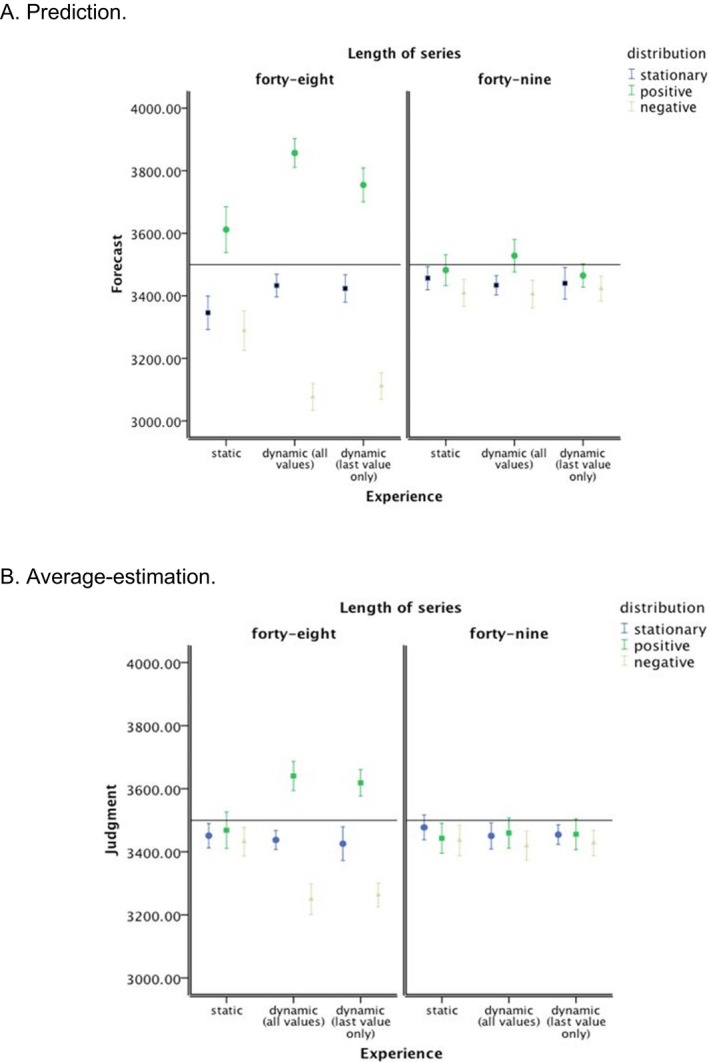
Means values for dependent variables in the experiment. Error bars represent 95% confidence interval of the mean.

The correlation between forecasting and estimation was low, *r *=* *.05, and non‐significant *p *>* *.05. The pattern of mean values for the two tasks (see Fig. 2A,B) indicates that forecasting performance was more influenced by trend direction than estimation performance and this influence was most pronounced under dynamic presentation (historic or momentary) and with consistent trends. In particular, the forecasting response seemed to be more strongly influenced by the final values in the series than the estimation response. Thus, performance on the two tasks was genuinely different and further analysis in terms of error of forecasting and average estimation was subsequently conducted. Furthermore, the pattern of results is consistent with the idea that under static presentation, the whole presented series is taken into account to a larger extent when a response is made. In contrast, under (in particular historic‐) dynamic presentation of consistent trend series, the most recent value in the series is more heavily weighted in the behavioral forecasting and estimation of the average responses.

#### 3.2.2 Forecasting

**Figure 3:**
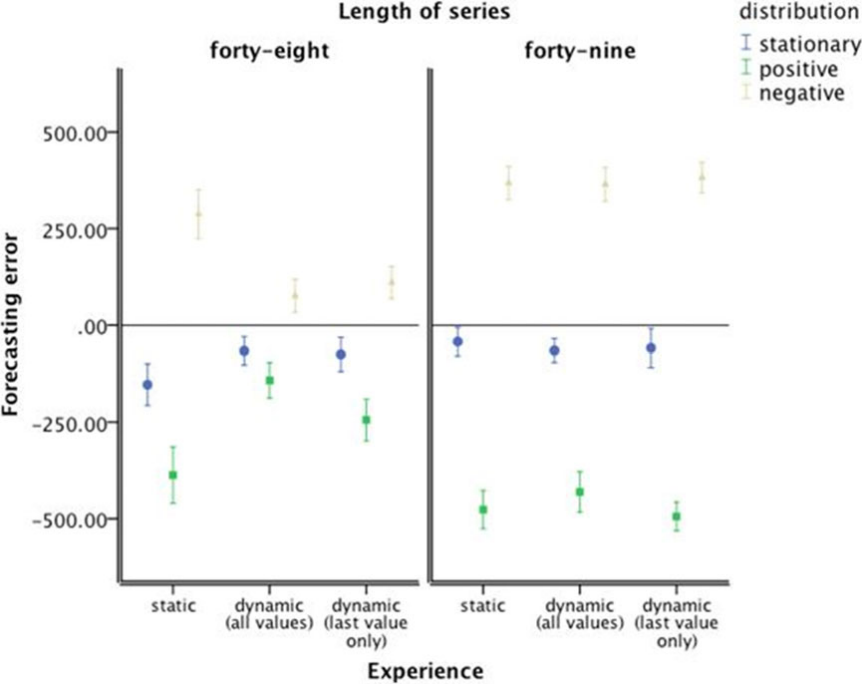
Prediction error. Error bars represent 95% confidence interval of the mean.

Overall, the following behavioral forecasting results reveal that dynamic presentation aids the forecasting accuracy of consistent trends. Fig. 3 shows the prediction error (calculated as the difference between response value and predicted next trend value from the regression equation of the trend that was presented) and illustrates that forecasting was more accurate with dynamic presentations than with static presentation. One‐way analysis of variance (anova) comparing the mean absolute error rate in the forecasts confirmed a significant difference between the three presentation conditions, *F*(2, 1617) = 18.08, ε^2^ = .02, *p *<* *.001. Post hoc tests (Tukey's HSD) confirmed that participants experiencing historic‐dynamic presentation series were significantly more accurate forecasters than participants experiencing static presentation (95%‐confidence interval of mean difference = [51.49; 117.50]) or momentary‐dynamic presentation (95%‐confidence interval = [13.20; 79.21]).

Trend‐damping was evident for each presentation mode—future salary was under‐predicted for a positive trend and over‐predicted for a negative trend. However, these tendencies were both markedly reduced with dynamic presentation in consistent trends, resulting in improved forecasting with the dynamic presentation mode (see Fig. 3). A 3 × 3 × 2 anova (Table 1, Panel 1) on the signed forecast errors confirmed that the effect of trend direction and the interaction effect of trend direction and presentation mode on forecasting error were statistically significant, but—unsurprisingly—given the net effect of positive and negative errors, the main effect of presentation mode was not. However, the interaction effect of trend consistency and trend direction was also significant, with the effect of direction stronger for inconsistent trends, as was the three‐way interaction of trend direction, presentation mode, and trend consistency with the interaction effect of direction and presentation mode only apparent for consistent trends.

**Table 1:** Analysis of variance—prediction error

Source	*df*	*F*	*p*	ε^2^
Panel 1: 3 × 3 × 2 analysis, all conditions				
Presentation mode	2	1.64	.194	.00
Direction	2	1,029.76	.000	.51
Length	1	1.93	.165	.00
Presentation by direction	4	14.49	.000	.01
Presentation by length	2	0.75	.474	.00
Direction by length	2	118.06	.000	.06
Presentation by direction by length	4	11.85	.000	.01
Residual	1,602			
Total	1,619			
Panel 2: 3 × 3 analysis, inconsistent trend				
Presentation mode	2	0.28	.756	.00
Direction	2	1,120.04	.000	.73
Presentation by direction	4	1.24	.292	.00
Residual	801			
Total	809			
Panel 3: 3 × 3 analysis, consistent trend				
Presentation mode	2	1.83	.161	.00
Direction	2	194.65	.000	.30
Presentation by direction	4	21.45	.000	.07
Residual	801			
Total	809			
Panel 4: One‐way analysis, consistent trend, static presentation				
Direction	2	114.00	.000	.46
Residual	267			
Total	269			
Panel 5: One‐way analysis, consistent trend, momentary‐dynamic presentation (last value only)				
Direction	2	27.49	.000	.17
Residual	267			
Total	269			
Panel 6: One‐way analysis, consistent trend, historic‐dynamic presentation				
Direction	2	56.27	.000	.29
Residual	267			
Total	269			
Panel 7: One‐way analysis, consistent trend, stationary series				
Presentation mode	2	4.41	.013	.03
Residual	267			
Total	269			
Panel 8: One‐way analysis, consistent trend, positive trend				
Presentation mode	2	17.29	.000	.11
Residual	267			
Total	269			
Panel 9: One‐way analysis, consistent trend, negative trend				
Presentation mode	2	20.20	.000	.13
Residual	267			
Total	269			

Given the significant three‐way interaction, follow‐up 3 × 3 anovas analyzed the effect of trend direction and presentation on forecasting error for each trend consistency. For inconsistent trends (Table 1, Panel 2), the main effect of trend direction was significant, but the main effect of presentation mode and the interaction effect were not. For consistent trends (Table 1, Panel 3), the effect of trend direction, *F*(2, 801) = 194.65, ε^2^ = .30, *p* < .001, and the interaction effect of trend direction and presentation mode on forecasting error were again statistically significant, but—unsurprisingly—given the net effect of positive and negative errors, the main effect of presentation mode was not. Simple effect tests (univariate anova) showed that with consistent trends the effect of trend direction was significant for both static (Table 1, Panel 4) and dynamic presentations, though with a smaller effect size for the latter (see Table 1, Panel 5 [momentary‐dynamic] and Table 1, Panel 6 [for historic‐dynamic]).

Further simple effect tests showed that with consistent trends the effect of presentation mode on forecasting error was significant for the stationary series (Table 1, Panel 7), for the positive trend (Table 1, Panel 8) and for the negative trend (Table 1, Panel 9). Post hoc tests (Tukey's HSD) corroborated the behavioral advantage (forecasting accuracy) with experience of dynamic presentation modes. Compared to the static presentations, the dynamic presentations significantly reduced under‐predicting of positive trends (95%‐confidence interval of mean difference = [−343.73; −146.38] for historic‐dynamic, and 95%‐confidence interval = [−241.55; −44.19] for momentary‐dynamic) and over‐predicting of negative trends (95%‐confidence interval = [127.54; 296.01] for historic‐dynamic and 95%‐confidence interval = [92.90; 261.37] for momentary‐dynamic).

These results (see also Fig. 3) demonstrate more behavioral adjustment toward the final (and most recent) values in the conditions where the trend series are dynamic and consistent. Specifically, the increase in forecasting accuracy for dynamic presentation is due to reduced trend‐damping; the dynamic presentation of trend series draws forecasters' attention to a smaller subset of more recently experienced events. For trended series, more recent events are closer to the normatively correct value than less recent events, so anchoring on more recent values would result in more accurate forecasts.

#### 3.2.3 Average‐estimation

**Figure 4:**
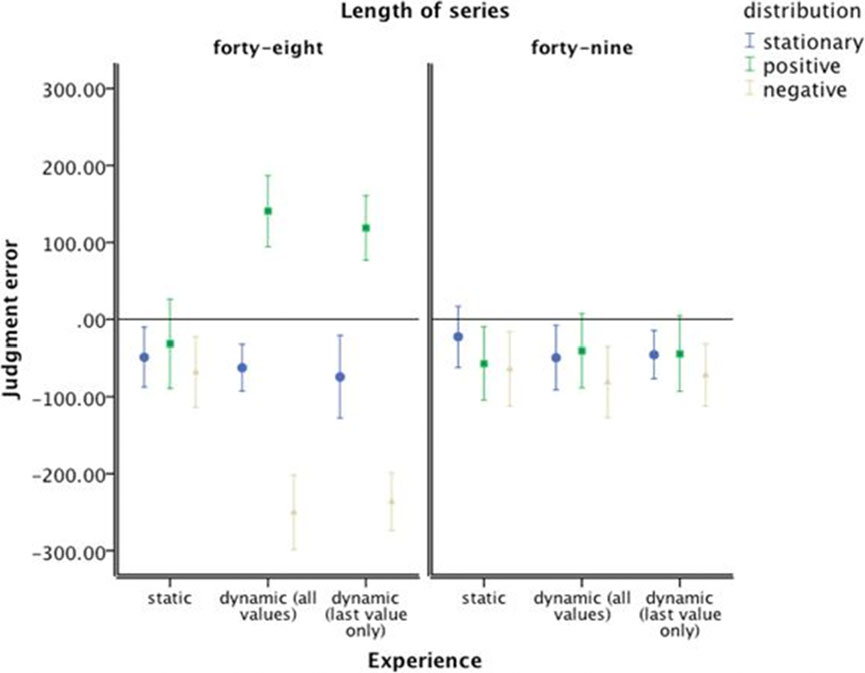
Average‐estimation error. Error bars represent 95% confidence interval of the mean.

In contrast to the behavioral forecasting results, the average‐estimation judgments results reveal that static presentation aids the average‐estimation accuracy of consistent trends. Average salary was generally underestimated (Fig. 4), but not for the consistent positive trends with dynamic presentation. One‐way anova comparing the mean absolute error rate in average estimation demonstrated no significant difference between the three presentation modes, *F *<* *1.

With dynamic presentation, there was a difference in the signed error between the positive and negative trended series, suggesting again anchoring on a subset of more recent events. A 3 × 3 × 2 anova (Table 2, Panel 1) on the signed average‐estimation errors confirmed that the effect of trend direction and the interaction effect of trend direction and presentation mode on error in the judged average value were statistically significant. Unsurprisingly, given the net effect of positive and negative errors, the main effect of presentation mode was not. However, the interaction effect of trend consistency and trend direction was also significant, with the effect of direction stronger for consistent trends. Moreover, the three‐way interaction of trend direction, presentation mode, and trend consistency was significant too, with the interaction effect of direction and presentation mode only apparent for consistent trends.

**Table 2:** Analysis of variance—average‐estimation error

Source	*df*	*F*	*p*	ε^2^
Panel 1: 3 × 3 × 2 analysis, all conditions				
Presentation mode	2	0.36	.695	.00
Direction	2	61.45	.000	.06
Length	1	0.14	.705	.00
Presentation by direction	4	11.30	.000	.02
Presentation by length	2	0.08	.919	.00
Direction by length	2	42.40	.000	.04
Presentation by direction by length	4	8.02	.000	.02
Residual	1,602			
Total	1,619			
Panel 2: 3 × 3 analysis, inconsistent trend				
Presentation mode	2	0.13	.877	.00
Direction	2	1.85	.157	.00
Presentation by direction	4	0.30	.878	.00
Residual	801			
Total	809			
Panel 3: 3 × 3 analysis, consistent trend				
Presentation mode	2	0.31	.733	.00
Direction	2	99.20	.000	.18
Presentation by direction	4	18.50	.000	.07
Residual	801			
Total	809			
Panel 4: One‐way analysis, consistent trend, static presentation				
Direction	2	0.58	.561	.00
Residual	267			
Total	269			
Panel 5: One‐way analysis, consistent trend, momentary‐dynamic presentation (last value only)				
Direction	2	62.37	.000	.32
Residual	267			
Total	269			
Panel 6: One‐way analysis, consistent trend, historic‐dynamic presentation				
Direction	2	83.53	.000	.38
Residual	267			
Total	269			
Panel 7: One‐way analysis, consistent trend, stationary series				
Presentation mode	2	0.37	.692	.00
Residual	267			
Total	269			
Panel 8: One‐way analysis, consistent trend, positive trend				
Presentation mode	2	14.38	.000	.09
Residual	267			
Total	269			
Panel 9: One‐way analysis, consistent trend, negative trend				
Presentation mode	2	20.95	.000	.13
Residual	267			
Total	269			

Given the significant three‐way interaction, follow‐up 3 × 3 anova s analyzed the effect of trend direction and presentation on error in the judged average value for each trend consistency. For inconsistent trends (Table 2, Panel 2), neither the main effect of trend direction nor the main effect of presentation mode nor the interaction effect was significant. For consistent trends (Table 2, Panel 3), the effect of trend direction and the interaction effect of trend direction and presentation mode were again statistically significant, but—unsurprisingly—given the net effect of positive and negative errors, the main effect of presentation mode was not. Simple effect tests (univariate anova) showed that with consistent trends the effect of trend direction on error in the judged average value was not significant for static presentation (Table 2, Panel 4), but was significant for both dynamic presentations—momentary‐dynamic (Table 2, Panel 5) and historic‐dynamic (Table 2, Panel 6).

Further simple effect tests showed that with consistent trends the effect of presentation mode on error in the judged average value was significant for the positive trend (Table 2, Panel 8) and the negative trend (Table 2, Panel 9), but not for the stationary series (Table 2, Panel 7). Post hoc tests (Tukey's HSD) corroborated the behavioral advantage (accuracy of average estimation) with experience of static presentation mode. Compared to the dynamic presentations, static presentations significantly reduced over‐estimation of positive trends (95%‐confidence interval of mean difference = [−254.34; −89.68] for historic‐dynamic, and 95%‐confidence interval = [−232.61; −67.95] for momentary‐dynamic) and under‐estimation of negative trends (95%‐confidence interval = [108.39; 256.19] for historic‐dynamic and 95%‐confidence interval = [94.58; 242.38] for momentary‐dynamic).

## 4 Model assessment

### 4.1 How the ADAM predicts behavioral forecasting and average‐estimation

The task‐specific subsystems of ADAM were implemented in a MATLAB program (http://uk.mathworks.com) that takes experimentally presented sequences (3)–(5) as inputs and produces behavioral responses as outputs. The model performance is thus directly comparable to the human data and is tested in a simulation experiment that replicates the behavioral experiment.

We statistically tested model predictions against the response data. First, in the case when *k*_*p*_ = 0 (without Gaussian noise), the model is deterministic and generates constant predictions as *BR*_*ik*_ is calculated according to (2). We note that in the case of forecasting under dynamic presentation we have the last element of the experimental sequence as a model prediction and in the case of average‐estimation judgment under static presentation the model predicts the mean of the experimental sequence as a behavioral response. Second, in the case when *k*_*p*_ ≠ 0, the model takes into account individual variability. Hence, we can simulate the responses given in the experiment better and compare the average predicted in the model with the experimentally observed averages for each experimental condition. In our simulations, we used *k*_*p*_ = .04 as estimated in the memory‐based scaling model (Petrov & Anderson, 2005). For each experimental condition (*n *=* *90) and each of two values of the model parameter *k*_*p*_ (0 and .04), we generated 90 model predictions that were to be compared with the 90 actual response data in that condition.

**Figure 5:**
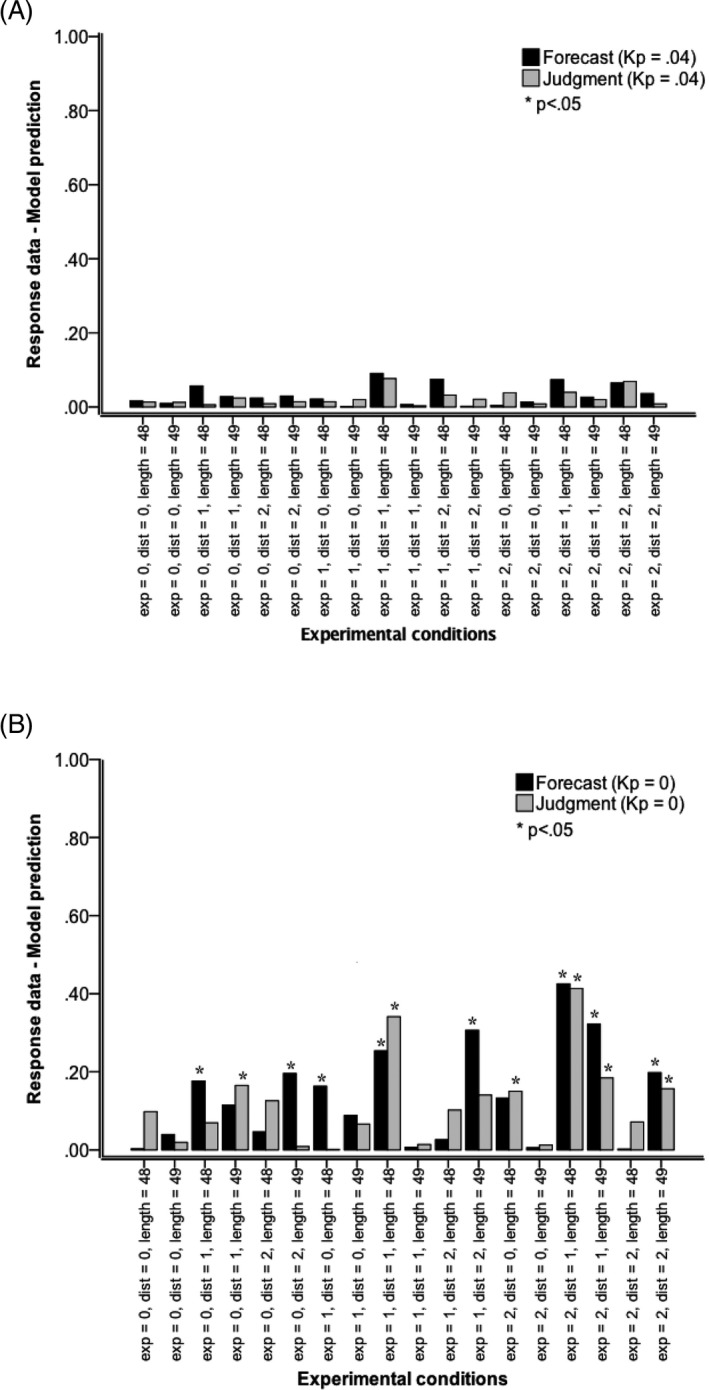
Effect size *r* of comparisons between response data and model predictions (ADAM). (A) The model simulations were performed with perceptual noise parameter *k* = 0.04 and (B) the model simulations were performed with perceptual noise parameter *k* = 0. Model comparisons: Exp (presentation mode): 0 static (one simultaneous presentation of all values of a series]), 1 historic‐dynamic [self‐paced value‐by‐value presentation of a series with all previous values visible at all times], and 2 momentary‐dynamic (self‐paced value‐by‐value presentation of a series with only the last value visible at any one time). Dist (trend direction): 0 stationary series (Gaussian noise only), 1 positive linear trend with superimposed Gaussian noise, and 2 negative trend with superimposed Gaussian noise. Length (trend consistency): 48 according to Eqs. (1), (2), (3) or 49, where the mean value of 3,500 was inserted at the end of a series of 48—an inconsistent continuation of positive and negative trends.

Unrelated *t* tests were used to assess the difference ([lack of] fit) between response data and model (ADAM) predictions. Fig. 5 shows the effect sizes for different combinations of trend direction, presentation mode, and trend consistency. With *k*_*p*_ = 0, 8 out of 18 differences were significant for forecasting, as were 6 of 18 for judgment. According to Cohen's (1988) conventions for effect size *r*, average effect size was small (*M* = .14 [*SD* = .13] for forecasting and mean = .12 [*SD* = .11] for average estimation), but not negligible and indicating a lack of fit between response data and model. For *forecasting*, model fit was poorest in experimental conditions with momentary‐dynamic presentation of positive trend and negative inconsistent trend, and historic‐dynamic presentation of positive and negative consistent trend. For *judgment*, fit was poorest in experimental conditions with (historic‐ or momentary‐) dynamic presentation of consistent positive trend.

With *k*_*p*_ = .04, none of the differences was significant, all *p *>* *.05 (and *|t|* < 1 for 16 out of 18 combinations). Average effect size was extremely small and consistent across the 18 experimental conditions, *M* = *SD* = .03, for forecasting, *M* = *SD* = .02, for judgment, indicating an excellent fit. The source of the data (our human participants or ADAM) explained hardly any variance: <.01% of variance in forecasting data and <.05% of variance in average estimation data—again evidence for a good fit.

Further evidence for the advantage for the simulation with model parameter *k*_*p*_ = .04 comes from comparisons between simulations with *k*_*p*_ = .04 and those with *k*_*p*_ = 0. Of the 14 conditions where the mean of the response data differed significantly from the mean of the simulation results with *k*_*p*_ = 0, 10 conditions also showed a significant difference between the simulation results with *k*_*p*_ = .04 and those with *k*_*p*_ = 0 at the .05 significance level, and another three conditions showed a significant difference at the .10 level.

An explanation for the difference in results is that the model with *k*_*p*_ = .04 represents a degree of individual variability, whereas model with *k*_*p*_ = 0 represents no variability at all. In fact, in the current dataset Petrov and Anderson's (2005) parameter value *k*_*p*_ = .04 produced variability in the series of model predictions that was very close to variability in the series of the actual data, mean of *SD* ratio = 1.00 (*SD* of *SD* ratio = 0.12) for forecasting and mean of *SD* ratio = 1.00 (*SD* of *SD* ratio = 0.11) for average estimation. Further confirmation of this finding comes from the Levene's test results showing that the assumption of equality of variance was not violated in any of the 18 experimental conditions. Given that fit between model and data was substantially better with *k*_*p*_ = .04 compared to *k*_*p*_ = 0 (see also Fig. 5), *k*_*p*_ = .04 was used in the following assessment of model predictions.

Cross‐validation within the large sample (*N *=* *1,620) was undertaken by randomly splitting the sample for each experimental condition (*n* = 90) in two subsamples (45 + 45). 2 × 2 anova was then conducted with independent variables series (response data or model predictions) and subsample for both tasks. The main purpose of the analysis was, first, to establish that the interaction effect was not significant (showing that the effect of series, and thereby fit between response data and model predictions, was not different between the two samples) and that the main effect of series was not significant (indicating a good model fit). For none of the experimental conditions were the interaction effect or the main effect of series significant, all *p *>* *.05, thereby providing evidence for consistency of model fit across subsamples within the dataset.

### 4.2 Predicting behavioral response with a linear‐regression time series model

**Figure 6:**
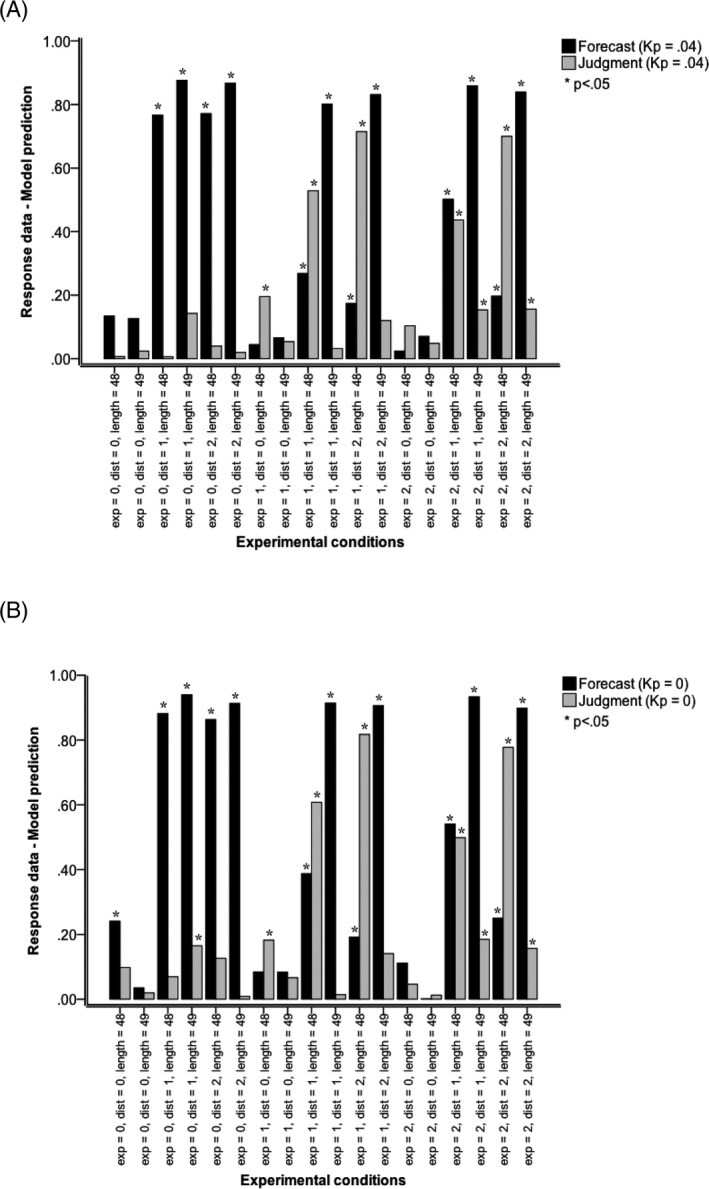
Effect size *r* of comparisons between response data and model predictions (Linear‐regression time series model). (A) The model simulations were performed with perceptual noise parameter *k* = 0.04 and (B) the model simulations were performed with perceptual noise parameter *k* = 0. Model comparisons: Exp (presentation mode): 0 static (one simultaneous presentation of all values of a series), 1 historic‐dynamic (self‐paced value‐by‐value presentation of a series with all previous values visible at all times), and 2 momentary‐dynamic (self‐paced value‐by‐value presentation of a series with only the last value visible at any one time). Dist (trend direction): 0 stationary series (Gaussian noise only), 1 positive linear trend with superimposed Gaussian noise, and 2 negative trend with superimposed Gaussian noise. Length (trend consistency): 48 according to Eqs. (1), (2), (3) or 49, where the mean value of 3,500 was inserted at the end of a series of 48—an inconsistent continuation of positive and negative trends.

Model fit was further assessed through comparison by way of statistical testing of the predicted next trend value against response data. As a comparison, the same type of analysis was conducted comparing response data with the predicted next trend value (see Fig. 6). To represent individual variability (*IV*) in task performance in the same way as in the model with *k*_*p*_ = .04, the predicted value was weighted by a random process as in Eq. (2):

(6) $$PV_{ik}\left(t\right)=Y\left(t\right)\times \left(1+k_{p}\xi_{p}\right)$$

Fig. 6 shows the effect sizes for different combinations of trend direction, presentation mode, and trend consistency. Twelve out of 18 differences were significant for forecasting and 7 of 18 for average estimation. Average effect size was large for forecasting, *M* = .46, *SD* = .36, and mid‐sized for average estimation, *M* = .19, *SD* = .23, indicating a poor fit and a lack of consistency of fit across experimental conditions. For *forecasting*, fit was extremely poor in experimental conditions with static presentation of positive or negative trends, historic‐dynamic presentation of positive inconsistent and negative inconsistent trends, and momentary‐dynamic presentation of positive inconsistent and negative inconsistent trends. Fit was also poor for momentary‐dynamic presentation of consistent positive and negative trends, and dynamic‐historic presentation of the consistent positive trend. For *average estimation*, fit was extremely poor in conditions with (historic‐ or momentary‐) dynamic presentation of the negative trend. Fit was also poor for historic‐ and momentary‐dynamic presentation of the consistent positive trend as well as historic‐dynamic presentation of the stationary trend. These results demonstrate that a linear‐regression time series model that describes the trend that is presented and generates the predicted next trend value is a poor model of people's response. In addition, it is notable that even the model of (2) with *k*_*p*_ = 0 performed better than the predicted next trend value, the model of (6) (compare Fig. 5 with Fig. 6).^2^

As a further comparison, the same type of analysis was conducted comparing model predictions of (2) with the model predictions of (6), the predicted next trend value (see Fig. 6). The results were almost identical to those of the previous comparison between the response data and the model predictions from (6), thereby indirectly providing further evidence for the excellent fit between the response data and model predictions of (2) and a poor fit between the response data and model of predictions of (6).

### 4.3 Predicting behavioral forecasting with ARIMA and exponential‐smoothing algorithms

Previous research (e.g., Andreassen & Kraus, 1990; Box, Jenkins, & Reinsel, 1994; Bunn & Wright, 1991; Gardner, 1985; Gardner & McKenzie, 1985; Lawrence, Edmundson, & O'Connor, 1985; Lawrence & O'Connor, 1992, 1995) has used ARIMA and exponential‐smoothing algorithms to explore and model people's forecasting behavior. We therefore compared these algorithms with ADAM. We statistically tested model predictions from an ARIMA (1, 0, 0) model (first‐order autoregressive model, also known as Box–Jenkins model [Box et al., 1994]) and those from an exponential‐smoothing algorithm (Gardner, 1985; Gardner & McKenzie, 1985) against the response data. For each experimental condition (*n *=* *90), we generated 90 model predictions from Eqs. (3) (for an increasing trend), 4 (for a decreasing trend), or 5 (for a stationary trend) that were to be compared with the 90 actual response data in that condition.

**Figure 7:**
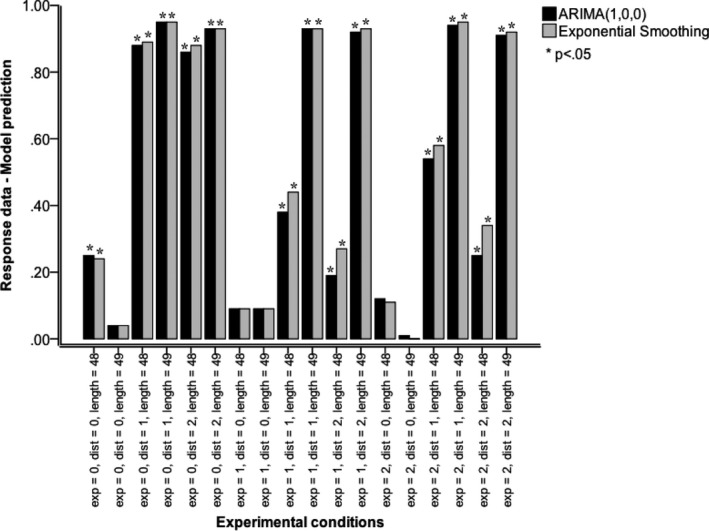
Effect size *r* of comparisons between response data (forecasting only) and model predictions (ARIMA and exponential smoothing models). Model comparisons: Exp (presentation mode): 0 static (one simultaneous presentation of all values of a series), 1 historic‐dynamic (self‐paced value‐by‐value presentation of a series with all previous values visible at all times), and 2 momentary‐dynamic (self‐paced value‐by‐value presentation of a series with only the last value visible at any one time). Dist (trend direction): 0 stationary series (Gaussian noise only), 1 positive linear trend with superimposed Gaussian noise, and 2 negative trend with superimposed Gaussian noise. Length (trend consistency): 48 according to Eqs. (1), (2), (3) or 49, where the mean value of 3,500 was inserted at the end of a series of 48—an inconsistent continuation of positive and negative trends.

Fig. 7 shows the effect sizes for different combinations of trend direction, presentation mode, and trend consistency. For ARIMA(1, 0, 0), 13 of 18 differences were significant for forecasting. The average effect size *r* was medium (*M* = .51 [*SD* = .39]), indicating a lack of fit between response data and model. Model fit was poorest in experimental conditions with momentary‐dynamic or historic‐dynamic presentation of positive and negative trends or inconsistent trends, and with static presentation of consistent or inconsistent positive or negative trends. For exponential smoothing the pattern of results was the same, with the same 13 out of 18 differences significant for forecasting. The average effect size *r* was medium (*M* = .53 [*SD* = .39]), indicating a lack of fit between response data and model. Model fit was poorest for the same conditions as with ARIMA(1, 0, 0). In sum, ARIMA and exponential‐smoothing models poorly fit our behavioral response data.

### 4.4 Model comparisons: ADAM, ARIMA, and exponential smoothing

Next, we statistically compared model predictions of ADAM, ARIMA(1, 0, 0), and exponential smoothing. We found that, overall, ADAM's prediction differs significantly from those of ARIMA(1, 0, 0), *t*(3238) = −2.86, *p *<* *.01, *r *=* *.05, and exponential smoothing, *t*(3238) = −2.74, *p *<* *.01, *r *=* *.05, but ARIMA and exponential smoothing do not differ significantly, |*t*| < 1, *r *=* *.00. The biggest differences between ADAM and ARIMA(1, 0, 0) were observed for static presentation of both consistent and inconsistent increasing and decreasing trends, and for historic and momentary dynamic presentation of inconsistent increasing and decreasing trends.

As a further comparison, model predictions of ARIMA(1, 0, 0) and exponential smoothing were compared, with the predicted next trend value from a linear‐regression time series model. The results show that, overall, the prediction of neither of the model differs significantly from the predicted next trend value |*t*| < 1. In sum, the predictions of ARIMA and exponential‐smoothing models differ substantially from ADAM's predictions, but not from those of a linear‐regression time series model.

## 5 General discussion

Many problems in the world confronting us are related to forecasting and anticipating events whose actual outcomes have not been observed yet. Although forecasters sometimes review time series retrospectively, commonly forecasters will experience a time series of events dynamically in real time. Certainly, most informal everyday forecasting where we anticipate such things as the daily moods of our boss, our favorite team's next game, or the price of beer will be based on dynamically experienced time series. However, all the studies of judgmental time series forecasting of which we are aware investigate how, when presented with a series of data points, forecasters predict the next event(s) in the presented series. Specifically, behavioral forecasting time series studies (cf. Lawrence & O'Connor, 1992) used procedures whereby the events were presented to participants as a static time series and respondents engage in multiple forecasts. In contrast, our method employs both dynamic and static presentations of time series and assesses how the accuracy of human forecasting and judgment depends on the presentation mode (dynamic and static) that is experienced and type of judgment (forecasting and estimation of the average) in “one‐shot” forecast and judgment.

Accordingly and consistent with our results, a number of authors have argued and empirically established that better probabilistic judgments are made through trial‐by‐trial experience (Gigerenzer, Hell, & Blank, 1988; Koehler, 1995; Spellman, 1996). However, experience did not only influence one type of judgment. We found that judgments in dynamic mode were different from those in static mode; specifically, they were anchored on more recent events for both types of judgment (forecasting and estimation of the average). One significant consequence was that forecasting accuracy was enhanced, but estimation of average worsened. The increase in forecasting accuracy for dynamic presentation is due to reduced trend‐damping; a possible explanation is that dynamic presentation draws attention to a smaller subset of more recent events. Accordingly, the experimental findings revealed a fourfold pattern of judgment accuracy, in which forecasting accuracy (where success accuracy is informed by the most recent event) is enhanced by dynamically experienced sequences of events but average estimation errors are induced because of recency. In contrast, where events are presented with static sequences (one simultaneous presentation of all values of a series), forecasting errors are induced (drawing the respondents' attention on the “average” event with insufficient adjustment to the most recent event) but average estimation accuracy is enhanced.

The modeling results revealed an excellent fit between response data and ADAM's predictions. With *k*_*p*_ = .04, none of the differences between response data and model (ADAM) predictions for both forecasting and judgment (average estimation) were significant. The results revealed evidence for the advantage for the simulation with model parameter *k*_*p*_ = .04 (representing a degree of individual variability). Given that fit between model and data was substantially better with *k*_*p*_ = .04 than with *k*_*p*_ = 0 (without Gaussian noise), *k*_*p*_ = .04 was used in further assessment of model predictions.

In addition, when model fit was further assessed, the results established that a linear‐regression time series model that describes the trend that is presented and generates the predicted next trend value is a poor model of respondents' forecasts and judgments. Similarly, the results also revealed a lack of fit between, on the one hand, Box–Jenkins (ARIMA; Box et al., 1994) and exponential‐smoothing models and, on the other hand, behavioral forecasting data. Moreover, we compared model predictions of ADAM, ARIMA, and exponential smoothing. We found that ADAM's prediction differs significantly from those of ARIMA and exponential smoothing, but ARIMA and exponential smoothing do not differ significantly. Moreover, ADAM accounts for the difference between processing sequences of dynamically and statically presented stimuli (visually presented data). In contrast to the ARIMA and exponential smoothing models, ADAM also accounts for behavioral judgment (non‐forecasting) tasks (e.g., average estimations). ADAM captured how variation in presentation mode produces variation in responses (and the accuracy of these responses) in both forecasting and judgment tasks.

The new ADAM proposed and tested in the present article embodies a number of principles for human judgments and forecasts: representations of time series and classification, similarity and psychologically weighted decision units, intrinsic variability, and recency. The representation subsystem accounts for presentation model and task in the judgment of perceptually encountered stimuli. This subsystem maps the external stimulus environment onto an internal decision space. Statistical analysis of this space explains why a simple behavioral response classifier is sufficient to account for the judgments and forecasts in our task. It is of course quite possible that other tasks might require more complex (e.g., nonlinear) decision classifications. Whether human perceptual judgments and forecasts may require a nonlinear decision classification is an important open empirical question largely unaddressed at present. Perhaps human subjects can explore complex perceptual decision spaces, in which case more complex multilayer models will be necessary. On the other hand, the behavioral response might indicate that the perceptual decision‐making system is restricted to simplified solutions even for problems that require nonlinear classification formulations for optimal performance.

Research on judgment and behavioral forecasting has argued that (a) rational and adaptive heuristics (e.g., Gigerenzer & Gaissmaier, 2011; Goldstein & Gigerenzer, 2009) or (b) “effort reduction” irrational heuristics (e.g., Payne, Bettman, & Johnson, 1993; Shah & Oppenheimer, 2008; Tversky, 1972; Tversky & Kahneman, 1974) underlie behavior (and accuracy) for judgments and forecasting. For example, Wübben and von Wangenheim (2008) explored the beneficial effect of recency (buying behavior) on forecasting accuracy. Relying only on recency heuristic (simple and more accurate than the Pareto negative binomial distribution model;^3^ also see Schmittlein, Morrison, & Colombo, 1987), forecasters are more accurate by ignoring information such as the frequency of previous purchases. Yet previous research did not explore systematically these assumptions across judgment and behavioral forecasting tasks, taking into account type of experience. Accordingly, in our argument, the way information is psychologically weighted depends on the (combination of) modes (presentation and task) in which the information that forms the basis for the judgment was acquired. Indeed, we found that human judgments are informed by presentation mode and judgment tasks with different consequences for prediction accuracy in behavioral forecasts and average estimations.

## 6 Conclusion

Given the theoretical and practical significance of the process and accuracy of people's judgment of numerical sequential information, future research should investigate how responses to experienced events and responses to described events differ in real‐life settings. Our model of adaptive anchoring (ADAM) provides a flexible vehicle for modeling this judgment, and we look forward to its wider application and further development to account for judgments across contexts.

## Notes

1 Typically, respondents are given a summary description of each option, including the likelihoods of the different payoffs.

2 Furthermore, as expected, the model of (2) performed worse when *k*_*p*_ = 0 instead of .04.

3 According to a Poisson process, in which purchases and dropouts are distributed according to a gamma distribution (Wübben & von Wangenheim, 2008).
